# Evaluation of Physicochemical Qualities and Trace Metal Levels of Barley and Malt in North and Central Gondar Zones, Ethiopia

**DOI:** 10.1155/ianc/7788608

**Published:** 2025-11-06

**Authors:** Mekuanint Lewoyehu, Bidir Kassaw, Tadesse Bizuayehu, Kenaw Gismie, Ashenafei Gezahegn

**Affiliations:** ^1^Department of Chemistry, Bahir Dar University, P.O. Box 79, Bahir Dar, Ethiopia; ^2^Department of Chemistry, Debark University, P.O. Box 90, Debark, Ethiopia; ^3^Department of Chemistry, University of Gondar, Gondar, Ethiopia; ^4^Department of Natural Resource Management, Debark University, Debark, Ethiopia

**Keywords:** flame atomic absorption spectrophotometer, permissible limit, quality analysis

## Abstract

Barley (*Hordeum vulgare*) is one of the earliest cereal crops cultivated in Ethiopia. Since barley and malt are widely consumed globally, conducting a spatiotemporal quality assessment is crucial to mitigate potential health risks for consumers. Therefore, this study investigated the physicochemical quality and the levels of selected metals in barley grown primarily for brewing purposes, as well as in the resulting malt, in the Gondar Zones of Ethiopia's Amhara Region. The investigation involved sample digestion with nitric and perchloric acids, and the resulting digestates were analyzed using an atomic absorption spectrophotometer equipped with a deuterium arc background corrector. The results indicate that, except for copper and nickel in barley grains, the concentrations of iron, zinc, and manganese in both barley and malt samples were approximately within the allowable limits established by the Food and Agriculture Organization (FAO) and the World Health Organization (WHO). The average contents in barley were 112.3 ± 7.3, 10.4 ± 1.7, 20.9 ± 3.0, 26.6 ± 2.3, and 11.6 ± 3.4 mg/kg for iron, copper, zinc, manganese, and nickel, respectively. The corresponding values in malt samples were 92.0 ± 5.9, 10.7 ± 4.2, 26.2 ± 5.2, 26.7 ± 6.2, and 18.0 ± 7.5 mg/kg, respectively. Although most trace metal levels and physicochemical qualities of barley and malt in this study fell within the acceptable ranges established by the European Brewing Convention (EBC), FAO, and WHO, continuous monitoring of trace metal levels in indigenous barley and its malt products is recommended to avoid potential health risks to consumers.

## 1. Introduction

Barley (*Hordeum vulgare*) is one of the oldest cultivated grains in the world, domesticated around 7000 BC [1, 2]. It is the fourth major staple crop globally, after maize, wheat, and rice, with an average yield of 2.89 t ha^−1^. In Ethiopia, barley ranks as the fifth most important crop, following teff (*Eragrostis tef*), maize, wheat, and sorghum, with an average yield of 2.5 t ha^−1^ [3–5].

Two distinct varieties of barley—food and malt—are cultivated in the country. Malt barley serves as the primary raw material for beer production in Ethiopia, accounting for 90% of production [6]. Despite the country's favorable environment and potential market opportunities, malt barley production remains relatively low, at approximately 15% of total barley production. Ethiopia has faced a persistent shortage of malt barley to meet the demands of local breweries [7]. An annual volume of roughly 130,000–150,000 metric tons (MT) would suffice to meet total domestic demand. Currently, local production covers only about 35% (45,500–52,500 MT) of this requirement, with the remaining 65% (85,000–97,500 MT) imported [8]. Therefore, expanding malt barley production is crucial to satisfy the growing demand and provide higher cash returns to farmers. Moreover, assessing the quality of barley is essential to ensure compliance with quality standards and to minimize health risks associated with the consumption of final products.

In Ethiopia, the Gondar malt factory serves as an important source of malt barley for nearby breweries [9]. The cultivation of this essential grain primarily occurs in the administrative woredas of Dabat, Janamora, Beyeda, Wogera, and Debark [9]. Trace metals can be introduced into both malt and barley during production and malting processes. Although small amounts of metals such as manganese, copper, and zinc are necessary micronutrients, excessive consumption can pose health risks to humans [10, 11]. Exposure to trace metals may occur through contaminated food and beverages, highlighting the importance of monitoring their levels in the human diet [12]. Saracoglu et al. [13] emphasized the need for regular monitoring of trace metal concentrations in human foods. Therefore, assessing the quality of barley and malt products is crucial to ensure the safety and quality of malt and the final beer, thereby protecting human health.

Several scholars [14–18] have investigated the chemical composition of barley across different regions. However, mineral contents vary depending on environmental conditions and soil types, resulting in significant variations in the chemical composition of cultivated barley. Therefore, area-specific studies are valuable to address the notable gap in the literature regarding the concentration of trace elements and the physicochemical quality of locally cultivated barley and its malt.

Considering this, we conducted the present study because no reports exist on the levels of trace elements in barley and malt in the aforementioned administrative regions. Filling these gaps is essential for ensuring food safety and informing evidence-based guidelines for the production and consumption of barley and malt products. Understanding the unique composition and concentrations of essential elements such as zinc, copper, iron, manganese, and nickel in locally cultivated barley and malt is crucial for assessing potential health implications and ensuring the safety of these staple foods. Moreover, the findings can inform crop cultivation practices, including fertilization strategies.

Therefore, the objective of this study was to assess the physicochemical qualities and the levels of selected trace elements in barley and malt samples from the specified administrative woredas. The findings may provide a foundation for developing evidence-based guidelines and strategies to manage and mitigate potential risks associated with trace metal exposure from the consumption of barley and malt. Furthermore, understanding the relationship between physicochemical characteristics and trace metal concentrations in these staple crops is essential for ensuring food safety and addressing concerns related to environmental and agricultural practices. The results can inform policies and practices that protect the health and well-being of communities that rely on these staple crops for sustenance.

## 2. Materials and Methods

### 2.1. Description of the Study Areas

The research area includes Beyeda woreda, which is approximately located at 13°30′0″ N latitude and 38°15′0″ E longitude, within the northern Gondar zone of the Amhara Regional State in Ethiopia. Janamora, also in the northern Gondar zone, is situated at approximately 13°6′0″ N latitude and 38°3′0″ E longitude. Debark woreda lies at 13°9′22″ N latitude and 37°53′53″ E longitude. Dabat, located in the northern Gondar zone, has a latitude of 12°59′3″ N and a longitude of 37°45′54″ E. Wogera, one of the prominent woredas in the central Gondar zone, is bordered by Mirab Bellessa to the south, Gondar Zuria to the southwest, Lay Armachiho to the west, Tach Armachiho to the northwest, Dabat to the north, Janamora to the northeast, and Misraq Bellessa to the southeast. The Wogera administrative center includes the towns of Gedebgie and Ameba Giorgis. The region supports the cultivation of various crops, including wheat, peas, beans, and barley, with commendable yields. Its suitability for malting barley production is attributed to the favorable low temperatures and its strategic geographic location within the research area.

The selection of the research areas—Beyeda, Dabat, Debark, Janamora, and Wogera woredas—from the Central and North Gondar zones was guided by several factors. First, barley is a key food crop in these zones in terms of area coverage, production, and overall importance. Second, the zones serve as micro-centers of barley diversity. Third, barley, being a short-season and early-maturing crop, is well suited to the cold climatic conditions that also favor grains such as wheat and teff. Fourth, the zones offer suitable temperatures, geological orientation, and favorable weather conditions for barley farming and malting.

**Figure 1:**
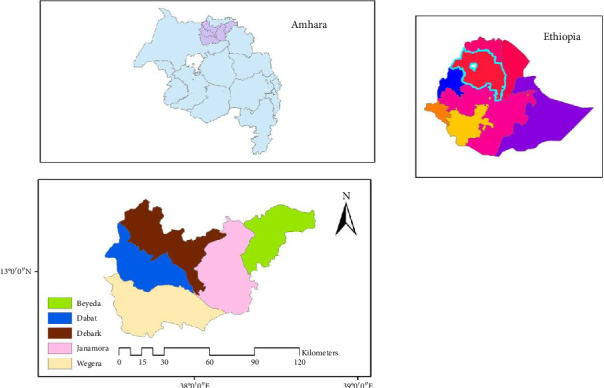
Map of the study areas.

The deliberate selection of these woredas is also due to their proximity to the Gondar Malt Factory, which relies on them as a major source of supply (Figure 1). Within these woredas, specific kebeles were chosen for the study based on their suitability for malting barley cultivation: Deresge, Woyina, and Inchetkab (Janamora); Adilemlem, Aterie, and Abarie (Beyeda); Adisgie Miligebsa, Kuha, and Gomia (Debark); Wokin, Amstugurare, and Talakmesik (Dabat); and Daber Lideta, Kossoye, and Dergaje (Wogera). These kebeles are considered ideal for malting barley due to their favorable low temperatures and strategic geographic locations within the study areas.

The research was conducted with the authorization of the University of Gondar, located in the Amhara Regional State of Ethiopia. Permission to collect samples was granted by the Gondar Malt Factory, allowing the study team to obtain malt samples during packing and barley samples as they were received from supply agencies. This collaboration between the academic institution and the industrial partner provided a solid foundation for a comprehensive investigation of the physicochemical qualities and trace element concentrations in locally cultivated barley and the resulting malt within the specified research areas.

### 2.2. Reagents, Chemicals, and Equipment

The malt and barley samples were analyzed using precise laboratory equipment. An electronic analytical balance (AA-200DS, Deriver Instrument Company, Germany) with a precision of ±0.0001 g was used to weigh the samples. The pH of barley and malt samples was measured using a pH meter (Elmetron CPI-501, Poland). Sample digestion was performed in a furnace (KDN-20C, China), and the digested samples were subsequently stored in a refrigerator (Beko RDP 6900, Japan) until analysis. Elemental concentrations of iron (Fe), zinc (Zn), manganese (Mn), copper (Cu), and nickel (Ni) were determined using a flame atomic absorption spectrometer (FAAS) (AA-500AFG, UK). The instrument was equipped with hollow cathode lamps and a deuterium background corrector and operated with an acetylene flame.

### 2.3. Sample Collection

Barley samples were collected from the Gondar Malt Factory, sourced from major barley-supplying districts, including Beyeda, Janamora, Debark, Dabat, and Wogera within the Central Gondar zone. A systematic random sampling approach was employed, involving the random selection of portions from different bags within the factory. Malt samples were collected concurrently during the packaging process using the same sampling method [19]. To ensure a representative and comprehensive analysis, the collected barley and malt samples were combined to form a pooled composite sample. The composite sample was thoroughly mixed using a homogenizer or sample divider to achieve uniformity [19]. Subsequently, the samples were securely stored in waterproof plastic containers and transported to the laboratory at the University of Gondar, where their physicochemical properties and trace metal concentrations were analyzed.

### 2.4. Physicochemical Properties and Procedures for Barley Quality Analysis

#### 2.4.1. Sieving

The sieving process began with the extraction of a precisely measured 100-g barley sample using a sample divider, ensuring an accuracy of 0.01 g. The sample was then placed on the top sieve, and the apparatus was operated for 5 min ± 10 s. Upon completion, the machine was opened to collect and examine four distinct fractions, specifically assessing broken kernels, grains of other cereals, and any foreign matter. Each barley fraction (I–IV) was carefully quantified, and the foreign matter content was expressed as a percentage of the total weight, rounded to the nearest 0.1%. The sum of fraction IV and the foreign matter constituted the rejected fraction [20].

#### 2.4.2. Moisture Content Determination

For moisture content determination, 4.5 g was taken from each sample and carefully weighed before being placed into a preweighed aluminum tray. The weight of each barley sample before and after drying at different sampling sites is provided in Supporting Table S5. The samples were then dried to a constant weight in an oven at 105.5°C for 3 h. After drying, the tray and its contents were transferred to a desiccator containing silica gel and allowed to cool for 20 min. The moisture content was subsequently calculated using the following formula [20]:

(1) $$\begin{array}{c} MC\left(\%\;\frac{w}{w}\right)=\left(\frac{\text{Mo}-\text{Mi}}{\text{Ms}}\right)\times 100\;\%, \end{array}$$

where MC is the moisture content, Mo is the mass of sample before drying, Mi is the mass of sample after drying, and Ms is the mass of sample taken.

#### 2.4.3. Thousand Kernel Weight (TKW)

The determination of TKW was carried out following standardized procedures. After the initial sampling using a sample divider, two separate lots of 40 g each were weighed. The weighing was performed with an electronic balance, ensuring precise measurement for the calculation of the weight of a thousand kernels.

(2) $$\begin{array}{c} \text{TKW}=\frac{W\;x\text{ DM }x\;10}{N}, \end{array}$$

where TKW is thousand kernel weight of dry barley/malt in grams, DM is dry matter (%*w*/*w*) of barley (100-MC), MC is moisture content, *W* is the weight of lots of barley/malt taken in grams, and *N* is the number of kernels in the lot taken.

#### 2.4.4. Germination Energy (GE): H_2_O Method

GE was assessed using the H_2_O method following a systematic procedure. Two separate lots of 100 kernels each were carefully selected using a sample divider, with half of the kernels and any foreign matter removed. The selected kernels were placed on a 90-mm filter paper in a petri dish. Subsequently, 4 mL of tap water was gently added to the dish, which was then covered with a lid to prevent evaporation [21]. Germination was carried out for 3–5 days in a controlled germination hood maintained at 18°C–20°C, allowing for observation of kernel growth. At 3 and 5 days after steeping, counts were made of nongerminated (*n*) and damaged (*d*) kernels. The results were reported based on these counts, providing insights into the germination energy of the barley kernels under the specified water-induced conditions.

(3) $$\begin{array}{c} \text{GE}\left(\frac{w}{w}\right)=100\%-\left(n+d\right). \end{array}$$

#### 2.4.5. Germination Capacity (GC): H_2_O_2_ Method

GC was assessed using the H_2_O_2_ method following a carefully calibrated procedure. A 0.75% H_2_O_2_ solution was prepared by diluting 5 mL of 30% H_2_O_2_ with 200 mL of distilled water. The solution was checked for concentration accuracy and stored in a refrigerator to maintain stability. Two separate lots of 200 kernels each were then selected using a sample divider, with half of the kernels and any foreign matter removed. The selected kernels were placed in a 200-mL conical flask and steeped in 200 mL of distilled water for 3–4 days at 18°C–20°C in a germination hood. After the steeping period, counts were made of nongerminated (*n*) and damaged (*d*) kernels. The results were reported based on these counts, providing insights into the germination capacity of the kernels under the specified H_2_O_2_-induced conditions [3].

(4) $$\begin{array}{c} \text{GC}\left(\frac{w}{w}\right)=\left[200-\frac{\left(n+d\right)}{2}\right]. \end{array}$$

#### 2.4.6. Protein Content

For protein content determination, a precise 600 g barley sample was measured using a beaker and then transferred into a grain analyzer (Perten Instrument). The analysis was performed using an infrared spectroscopic technique, which provides an accurate assessment of protein content and valuable insights into the nutritional composition of the barley. The use of advanced instruments such as the Perten analyzer enhances the precision and reliability of the protein analysis.

### 2.5. Physicochemical Properties and the Procedures for Malt Quality Analysis

#### 2.5.1. Friability Measurement

Friability was assessed using a 50-g barley sample. The malt sample was placed in a sieve drum, where a pressure arm pressed the malt against the sieve for 8 min [22]. During the measurement, both fully and partially unmodified malt grains were evaluated. Small fragments of physically modified material passed through the drum, while larger unmodified fragments were retained. The mass percentage (%w/w) of friability, specifically for the glassy (WUG and PUG) malt grains, was calculated after the 8-min milling process in the friability meter [23]. This method provides insights into the structural integrity and susceptibility to breakage of malt grains, offering valuable information for quality assessment in the brewing process.

#### 2.5.2. Acrospires Length

The length of acrospires in malt samples was assessed by extracting a sample from the germination box using a sample divider. A 5% hydrated copper sulfate (CuSO_4_•5H_2_O) solution was prepared by dissolving 50 g of CuSO_4_•5H_2_O in 1 L of water. For the analysis, 100 g of malt was carefully selected from the final polished malt and placed into a beaker. The malt was then boiled briefly with the CuSO_4_ solution until the acrospires became visible. During this process, the number of both modified and unmodified acrospires was counted. This method provides insights into the germination process and acrospire development, contributing to the overall quality assessment of the malt [24, 25]. The use of CuSO_4_ solution facilitates visualization and enumeration of acrospires, aiding in the evaluation of malt characteristics for brewing applications.

#### 2.5.3. Wort Preparation

The wort preparation process was conducted following meticulous procedures to ensure accurate and controlled conditions. The parameters, methods, and equipment used for the analysis of malt samples are provided in Supporting Table S1.

### 2.6. Malt Quality Analysis

#### 2.6.1. Milling

A quantity of 55 g of malt was milled, producing both coarse and fine particles, collected in a dry plastic box. The milled content was then emptied into dry beakers.

#### 2.6.2. Sample Weighing and Addition of Distilled Water

Malt sample (50 g) was precisely weighed. Distilled water (200 mL) was added to each of the fine and coarse samples.

#### 2.6.3. Congress Mashing Procedure

A laboratory-scale congress mashing program was employed, capable of producing 450 mL of wort per batch. Spent grain cylinders were placed into the holes of the mashing machine. Water levels in the machine reservoir were checked and adjusted with distilled water as necessary. The congress method was selected, and the machine was started, initiating the warming of the water reservoir to 45°C.

#### 2.6.4. Mashing Steps

Demineralized water (200 mL) was added to each cylinder, and the stirrers were activated to thoroughly mix the sample. The mash was initially held at 45°C for 30 min. The temperature was then gradually increased to 70°C at a rate of 1°C per minute over 25 min. Upon reaching 70°C, an additional 100 mL of demineralized water was added. The stirrers were rinsed with a small amount of water, and the exterior of the beaker was dried. The mash volume was finally adjusted to 450 ± 0.2 mL by adding distilled water.

#### 2.6.5. Filtration

The wort was filtered using fluted filter paper and a funnel. This procedure ensured the controlled and precise preparation of wort, a critical component in brewing, with careful attention to temperature, stirring, and volume adjustments [25].

#### 2.6.6. Saccharification Process

The saccharification process was carried out by maintaining the mash at 70°C for 1 h. During this period, the saccharification rate was monitored to track the enzymatic conversion of starches into sugars and dextrins. At regular intervals, a drop of mash was placed on a porcelain plate, followed by the addition of a drop of iodine solution 10 min after the addition of water. The iodine test was repeated at 5-min intervals until saccharification was complete. The appearance of a clear yellow spot indicated completion. The saccharification process is crucial in brewing as it reflects the efficiency of enzymatic conversion of malt starches into fermentable sugars. Monitoring the iodine test provides insights into the progression and completion of saccharification during the mashing stage [26].

#### 2.6.7. Filtration Process

The filtration process was conducted with precision and attention to detail: glass and plastic funnels were used in conjunction with pleated filter paper as the filtering material. Wort was collected into 500-mL vials. Wort was transferred directly from the standardized mashing cylinders onto the filters. The first 100 mL of wort was used to rinse the cylinders and brought back on top of the filter. This ensured complete filtration through the settled spent grains, utilizing them as a filter in addition to the fluted filter paper. Filtration continued until no wort drops emerged from the funnel into the vial. The speed of filtration was expressed as “Normal” if completed within 1 h. Filtration longer than 1 h was expressed as “Slow.” No other expressions were used.

#### 2.6.8. Organoleptic Assessment

The odor of the malt was assessed organoleptically. The odor was described as “caramel” when it matched the characteristic profile of the malt being analyzed. The extract content of the wort was determined according to the EBC method and calculated using the specified formulas (Equations (5) and (6)). This careful filtration procedure ensured effective separation of wort from spent grains, with attention to filtration rate and organoleptic properties. Recording filtration speed and evaluating malt odor provided valuable information for quality control in the brewing process [27].

(5) $$\begin{array}{c} E1\;\left(\%\frac{w}{w}\right)=\frac{\text{PX}\left(M+800\right)}{100-P}, \end{array}$$

(6) $$\begin{array}{c} E2\left(\%\frac{w}{w}\right)=E1\times 100\;\%\frac{1}{100-M}, \end{array}$$

where E1 is the extracted content of the sample in %w/w, E2 is the extracted content of dry malt in %*w*/*w*, *P* is the extracted content in wort in % (Plato) using table sugar, *M* is the moisture content of malt in %*w*/*w*, and 800 is the volume of distilled water (mL) added into the mash to 100 g of malt.

(7) $$\begin{array}{c} \text{Extract difference }\left(\%\right)=\text{Fine dry matter of extract sample}-\text{coarse dry matter of extract sample}. \end{array}$$

#### 2.6.9. Dynamic Viscosity (*ŋ*)

According to British standards, the dynamic viscosity of malt wort should be measured by using an outward viscometer, ensuring that the sample of particulate matter passes through a sintered glass filter (porosity). The viscometer was placed vertically in the water bath at 20°C and allowed to equilibrate for 30 min, and the flow time was measured between the fixed points of the outward viscometer after adding 10 mL of wort. Finally, the dynamic viscosity (*ŋ*) of malt was calculated using the following formulas [27].

(8) $$\begin{array}{c} ŋ\text{fast}=0.10221\left(2.221-ƿ\text{sample}\right)\times \text{tf},\\ ŋ\text{slow}=0.01024\left(2.220-ƿ\text{sample}\right)\times \text{ts}, \end{array}$$

where tf is the time of fast ball, ts is the time of slow ball, *ŋ*fast is the viscosity of fast, *ŋ*slow is the viscosity of slow, and ƿsample is the density of wort sample obtained by using an outward viscometer.

#### 2.6.10. pH

Five grams of wort were added to 35 mL of distilled water in a 250-mL beaker. The electrodes of a calibrated pH meter were immersed in the solution, and the pH was measured directly [28]. The appearance and odor of the wort were evaluated through direct observation. Physical assessments were conducted using the naked eye and nose to determine the visual characteristics and olfactory profile of the wort. This comprehensive evaluation combined quantitative (pH measurement) and qualitative (appearance and odor) assessments, providing a holistic understanding of the wort's chemical and sensory attributes.

### 2.7. Determining the Level of Trace Metals in Barley and Malt Samples

The levels of metals in barley and malt samples were analyzed with care to ensure the separation of the analyte from the interfering matrix, avoiding loss, contamination, or alteration of speciation, and minimizing analytical interference [19]. To optimize trace metal analysis, malt and barley samples were digested using a mixture of HNO_3_ and HClO_4_ under varying conditions, with minor procedural modifications [29]. Laboratory trials to determine the optimum conditions—including acid volume ratios, temperature, and digestion time—are presented in Supporting Tables S2 and S3 for barley and malt, respectively.

The optimized digestion method was applied to the samples for subsequent trace metal analysis. The optimal procedure was selected based on criteria such as clarity of digestion, minimal reagent consumption, efficient digestion time, and appropriate temperature—250°C for barley and 200°C for malt—ensuring complete digestion. The finalized procedure involved digesting 0.5 g of each sample with 6 mL of 69%–72% HNO_3_ and 2 mL of 70% HClO_4_. Digestion times were 1 h for barley and 2 h and 15 min for malt.

For example, the optimal condition for barley (entry No. 7, Supporting Table S2) involved digesting 1 g of sample with 6-mL HNO_3_ and 2-mL HClO_4_ at 250°C for 1 h, producing a clear, colorless solution. This condition was considered optimal due to complete digestion, the shortest processing time, moderate temperature, and efficient reagent usage. Similarly, the optimal condition for malt (entry No. 9, Supporting Table S3) involved digesting 1 g of malt with 6 mL HNO_3_ and 2 mL HClO_4_ at 200°C for 2 h and 15 min, yielding a clear, colorless solution within a short time, indicating complete sample digestion.

For the preparation of instrumental calibration standard solutions for FAAS, a stock standard solution (1000 mg/L) in 2% HNO_3_ containing trace metals (Fe, Zn, Mn, Cu, and Ni) was obtained from Buck Scientific Puro Graphic, USA. The accuracy of FAAS metal analysis is highly dependent on proper calibration and standard solution preparation procedures [9]. The operating conditions of the FAAS are provided in Table 1. Calibration curves generated from absorbance readings using five series of working standards are presented in Supporting Figure S1(A–E), corresponding to Fe, Cu, Zn, Mn, and Ni, respectively (Table 2).

The critical concentration of each metal was calculated using the regression equation *A* = *mC* + *b*, by solving for C at the minimum detectable absorbance of *A* = 0.004, which is a commonly accepted threshold for FAAS detection. For Zn, the calculated absorbance at *C* = 0 was 0.0154, which is higher than the detection threshold (*A* = 0.004), indicating that detection is feasible well below this threshold, and hence critical concentration is not applicable in the conventional sense. The critical concentrations were calculated as:

(9) $$\begin{array}{c} \text{Critical concentration}=\frac{\text{ Minimum absorbance}-b}{\text{slope}\left(m\right)}. \end{array}$$

To maintain the purity and integrity of the experimental process, distilled water with a high chemical purity (< 1.5 μS/cm) was consistently used for both sample preparation and dilution. All the plastic and glassware were cleaned with diluted HNO_3_ (5%) and rinsed with distilled water.

### 2.8. Method Validation and Quality Assessment

Method validation is a crucial step in ensuring the quality, reliability, and consistency of analytical results. The accuracy and precision of the analytical methods were evaluated through repeatability and recovery studies, specifically using matrix spike and laboratory control samples. Spike recovery helps assess potential losses of elements during sample digestion and subsequent processing. To determine Fe, Cu, Zn, Mn, and Ni levels in barley and malt samples, a spiking method was adopted, as Certified Standard Reference Materials (CSRM) were not readily available.

For recovery analysis, triplicate samples were spiked with known amounts of the target elements and subjected to the same digestion procedure as the actual samples. The response of the spiked sample was measured and compared to an identical spike in the standard diluent, and the percent recovery (%R) was calculated as follows:

(10) $$\begin{array}{c} \%R=\left[\frac{\text{Amount after spike}–\text{amount before spike}}{\text{Amount added}}\right]\times 100. \end{array}$$

Precision was assessed by calculating the relative standard deviation (RSD) of the triplicated results, where %RSD = (SD/mean value) × 100.

**Figure 2:**
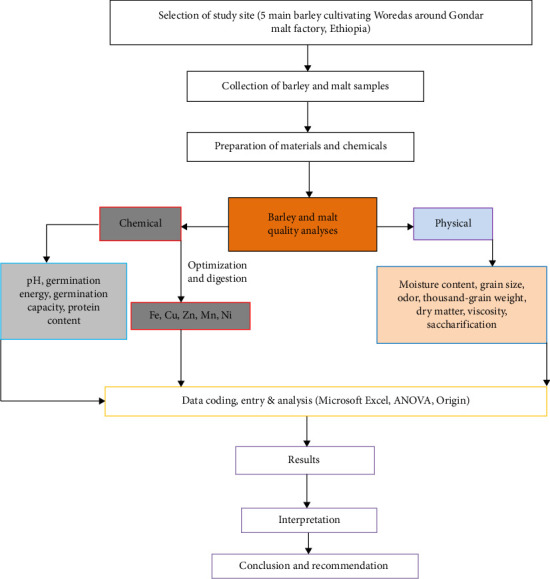
Methodological frameworks utilized for quality analysis of barley and malt.

Accuracy was expressed in terms of matrix spike recovery. Additionally, the analytical methods and the accuracy of the FAAS instrument for determining metal concentrations in barley and malt samples were validated by calculating the limit of detection (LOD) and limit of quantification (LOQ) using triplicate readings of the blank solution. The LOD and LOQ values for each metal are presented in Supporting Table S4. The overall methodological framework employed for the quality analysis of barley and malt samples is illustrated in Figure 2.

### 2.9. Statistical Analysis

A one-way analysis of variance (ANOVA) using SPSS version 22 was utilized to assess significant variations in trace metal levels of the samples collected from different sites. A probability level of *p* < 0.05 was considered statistically significant. Origin Pro 2018 was used to prepare calibration curves using absorbance readings of five series of standard solutions of each metal [27]. Data are presented as mean ± SD (standard deviation) (*n* = 3).

## 3. Results and Discussion

### 3.1. Physicochemical Qualities of Barley and Malt Samples

The physicochemical characteristics of barley and its malt products are influenced by several factors, including moisture content, GE, GC, protein content, TKW, friability, purity, grain size, homogeneity, wort viscosity, saccharification time, and wort filtration rate, among others. The following sections briefly discuss the key quality parameters of both barley grains and malt.

#### 3.1.1. Physicochemical Qualities of the Barley Samples

The moisture content of the examined barley samples ranged from 12.0% to 12.8% (Table 3), meeting the EBC system's recommended limit of ≤ 13.5% [12]. The GC test, used to evaluate barley grain dormancy, showed values ranging from 94% to 99% (Table 3), generally within the acceptable limit of ≥ 96%. According to EBC standards, the GC value should be ≥ 96%, and barley samples from Beyeda, Debark, and Dabat met this requirement, whereas those from Wogera and Janamora exhibited slightly lower values [22]. The slightly reduced GC in Wogera and Janamora barley may be attributed to elevated moisture levels during germination. GE, indicating the proportion of grains expected to fully germinate under normal malting conditions, ranged from 91% to 97% (Table 3), satisfying the EBC-recommended limit of ≥ 96% [25]. The protein content of the barley samples ranged from 8.4% to 9.9%, well within the EBC standard limit of ≤ 12%. TKW ranged from 37.2 to 39.5 g, consistent with the EBC standard (> 30 g). The purity percentage, another key parameter for assessing barley quality, varied from 96.1% to 98.3%, also meeting the EBC standard (≥ 96%). Debark exhibited the highest purity (98.3%), while barley samples from Beyeda, Wogera, Janamora, and Dabat showed comparable purity levels.

#### 3.1.2. Physicochemical Qualities of the Malt Sample

As mentioned earlier, malt quality is influenced by several factors, including growing conditions, temperature, moisture content, protein level, grain size, and the malting process [12]. Consistent with the findings of Kefale et al. [30], this study demonstrated the influence of both genetic and environmental factors on enhancing grain size. The results showed that 5.1% of the grains were larger than 2.2 mm, aligning with the EBC standard requirement of 3%–10% for grains > 2.2 mm (Table 4). Meeting the brewing industry standard, 93.6% of the grains in this study passed through 2.8- and 2.5-mm sieve sizes.

The moisture content of the malt samples (3.7%; Table 4) was within the acceptable EBC range of 3.5%–5.0% for barley malt. This value was notably lower than the industrial specification for safe storage (12.5%) [31]. The extract composition, a key parameter for mashing, also met EBC standards, with fine grind extract accounting for 79.1% and coarse grind extract for 76.4% of the total extract (Table 4). Other malt quality parameters—such as viscosity, odor, color, and TKW—were likewise consistent with EBC or industrial specifications (Table 4).

The saccharification time, representing the duration required for starch conversion to sugar during brewing, ranged from 10 to 15 min, in agreement with EBC standards. Extended saccharification periods typically indicate incomplete starch breakdown; however, all malt samples in this study met the required industrial quality.

The malt pH averaged 5.7 (Table 4), which falls within the EBC-specified range of 5.6–5.8 [22, 23]. This level of acidity is consistent with previous findings by Jones and Budde [32], who reported a pH range of 5.0–6.6. Maintaining pH within this range is essential for controlling microbial activity, particularly fermenting yeast, thereby ensuring wort quality.

The friability meter, which measures the physical disintegration of malt grains, recorded a value of 85%, exceeding the EBC standard requirement of > 80% for barley malt (Table 4), and thus meeting the highest industrial specification in Ethiopia.

### 3.2. The Level of Selected Trace Metals in Barley and Malt Samples

#### 3.2.1. Recovery Test Results

The percentage recovery test yielded values ranging from 91.4% to 106.3%, with a %RSD of < 10, confirming the quality, reliability, and consistency of the analytical results. Recovery values obtained from the barley samples ranged from 70.0% to 103.3% (Table 5), demonstrating that the analytical method met the required standards for both precision and accuracy. The highest recovery was recorded for Mn (103.3%), followed by Cu (92.9%) and Zn (91.0%), whereas Fe and Ni exhibited the lowest recoveries at 70.0% and 86.7%, respectively. Although most recoveries were within the acceptable range, the relatively low Fe recovery suggests possible analytical or matrix interference or losses during sample preparation. Notably, Cu, Mn, and Zn contents in the barley samples met the required standard limits, while Fe and Ni were below the specified thresholds. Overall, the recovery values (91.4%–106.3%) adhered to the FAO/WHO recommended standards, underscoring the reliability and accuracy of the analytical method. These findings affirm that the metal content analysis in barley samples was precise, consistent, and methodologically sound.

The mean concentrations of metals in barley and malt samples, along with their maximum permissible limits (MPLs) set by FAO/WHO [25], are presented in Table 6. The average metal concentrations in barley were 112.3 mg/kg for Fe, 10.4 mg/kg for Cu, 20.9 mg/kg for Zn, 26.6 mg/kg for Mn, and 11.6 mg/kg for Ni. In malt, the corresponding mean concentrations were 92.0 mg/kg for Fe, 10.7 mg/kg for Cu, 26.2 mg/kg for Zn, 26.7 mg/kg for Mn, and 18.0 mg/kg for Ni. These values provide insight into the metal composition of barley and malt, and their comparison with the FAO/WHO MPLs facilitates the evaluation of their safety and quality.

Barley from the Dabat district exhibited a notably higher Fe concentration than the other metals analyzed, with the highest Fe level (139.0 mg/kg) detected in Dabat, followed by Mn (30.0 mg/kg). Conversely, the lowest Cu level (6.5 mg/kg) was recorded in barley from Wogera. The highest Zn concentration was observed in Debark (28.0 mg/kg), followed by Beyeda (23.0 mg/kg), while the lowest Zn values were found in Janamora and Wogera (17.0 mg/kg). Overall, Fe concentrations in barley ranged from 70.5 to 139.0 mg/kg, with the highest value in Dabat and the lowest in Wogera.

The Cu content in barley samples ranged from 6.5 to 15.0 mg/kg, with Debark exhibiting the highest concentration (15.0 mg/kg), followed by Dabat (12.5 mg/kg), and Wogera the lowest (6.5 mg/kg). Although Cu is an essential micronutrient, excessive intake can be harmful; the average recommended daily intake is approximately 30 mg/day [28]. The Mn content ranged from 24.0 to 30.0 mg/kg, with the highest in Dabat and the lowest in Debark (Table 6). The mean Ni concentration varied from 9.0 to 15.0 mg/kg, with the highest value recorded in Beyeda and the lowest in Janamora. The intake of Ni through food depends on environmental and anthropogenic factors such as proximity to contamination sources. The Joint FAO/WHO Expert Committee on Food Additives established a tolerable daily intake of 5 mg/kg body weight for Ni; hence, the Ni levels observed in both barley and malt samples exceeded this limit.

A one-way ANOVA (Supplemental Tables S6–S10) revealed statistically significant differences among sites for Fe (*p* = 1.25 × 10^−5^), Cu (*p* = 0.0025), and Zn (*p* = 0.0088), indicating spatial variability in these metals. In contrast, Mn (*p* = 0.110) and Ni (*p* = 0.820) did not show significant variation, suggesting a relatively uniform distribution of these metals across the sampling sites. The observed variations in metal concentrations among districts are likely attributable to differences in soil composition and environmental conditions influencing metal uptake by barley. Conversely, the lack of significant variation in Mn and Ni may reflect similar soil chemistry for these elements across sites.

Importantly, the detection of all studied metals above the instrumental detection limits indicates that barley and its derived malt from the study areas could serve as potential dietary sources of these metals for consumers. Overall, the concentration patterns of metals varied among districts as follows: Beyeda: Cu < Ni < Mn < Zn < Fe; Janamora and Dabat: Ni < Cu < Zn < Mn < Fe; Debark: Ni < Cu < Mn < Zn < Fe; and Wogera: Cu < Ni < Zn < Mn < Fe.

Although various chemical investigations share similar objectives, discrepancies may arise due to differences in sampling procedures, sample preparation, and analytical techniques. Therefore, this study compared the concentrations of Fe, Cu, Zn, Mn, and Ni in barley and malt samples with those reported in previous studies, as summarized in Table 7.

The Ni content in the barley samples ranged from 8.17 to 15.0 mg/kg (Table 7), markedly higher than the ranges reported by Eticha and Hymete [33] (3.5–3.6 mg/kg) and Briggs [27] (3.4–4.2 mg/kg). Similarly, the Mn content in the present barley samples (24.3–28.9 mg/kg) exceeded the levels reported by Contreras-Jiménez et al. [34]. In contrast, the Zn concentrations in barley (17.9–23.9 mg/kg) were lower than those reported by Čejka et al. [25] (35.9–76.1 mg/kg) and Contreras-Jiménez et al. [34] (24.8–95.3 mg/kg).

Regarding malt, the Fe content measured in this study ranged from 86.6 to 97.9 mg/kg (Table 7), slightly higher than the values reported by Kumar et al. [35] (77.4–82.4 mg/kg). The Zn content in malt samples (20.9–31.4 mg/kg) was also marginally higher than the concentrations reported by Adams et al. [28] and Teixeira et al. [36]. While the Mn and Zn contents in barley were generally higher than those documented in previous studies [12, 37, 38], the Cu and Mn levels were comparable to earlier findings [39–42].

Notably, the concentrations of Cu, Zn, Mn, and Ni in malt were higher than those in barley. This increase may be attributed to the steeping and germination stages of malting, during which water containing trace amounts of these metals is used for cleaning and hydration. Consequently, a slight enrichment of metal content in malt compared to barley is expected.

## 4. Conclusions and Recommendations

This study investigated various physicochemical parameters and the levels of selected metals in barley and its malt. The findings revealed that the levels of iron, zinc, and manganese in both barley and malt samples generally complied with FAO/WHO limits, whereas copper and nickel exceeded the maximum permissible levels.

Although trace metal concentrations were largely within recommended limits, continuous monitoring of these elements in indigenous barley and malt is essential to prevent potential health risks associated with elevated metal levels. The implications of these findings extend beyond scientific interest to public health and agricultural management, providing a foundation for developing informed guidelines and strategies to mitigate trace metal exposure through barley and malt consumption.

The FAAS was less sensitive for detecting iron and nickel, underscoring the need for more advanced analytical methods in future studies. Regular monitoring of trace metal concentrations in barley and malt using more sensitive techniques is therefore strongly recommended. Such proactive efforts are vital to ensure the safety and quality of barley and malt products, particularly in regions with diverse environmental and agricultural conditions. Furthermore, this study contributes valuable insight into the safety and nutritional quality of barley and malt with respect to trace metal contamination—an issue of global concern amid rising industrialization and agrochemical use. These findings may also serve as a reference for countries with similar agro-ecological conditions and traditional food systems in establishing quality control measures and promoting food safety standards for cereal-based products.

## Figures and Tables

**Table 1:** The operating conditions of the flame atomic absorption spectrophotometer (FAAS) used in the analysis of the selected metals in the barley and malt samples.

Tested metal	Wavelength (nm)	Slit width (nm)	Lamp current (mA)	Energy (kJ/mol)
Fe	248.3	0.2	7.0	3.087
Cu	324.7	0.7	1.5	3.572
Zn	213.9	0.7	2.0	3.096
Mn	279.5	0.7	3.0	4.097
Ni	232.0	0.2	7.0	3.882

**Table 2:** Regression equations and the coefficient of determination (*R*^2^) values for each metal obtained from the calibration of the flame atomic absorption spectrophotometer (FAAS) employing five series of standard solutions of each metal.

Tested metal	Concentration of standards (μg/L)	Regression equation (*A* = *mC* + *b*)^∗^	Critical concentration (μg/L)	*R* ^2^
Fe	0.5, 1.0, 1.5, 2.0, 2.5, 3.0	*A* = 0.0075*C* − 0.0016	0.7467	0.9993
Cu	0.5, 1.0, 1.5, 2.0, 2.5, 3.0	*A* = 0.0113*C* + 0.0012	0.2478	0.9994
Zn	0.5, 1.0, 1.5, 2.0, 2.5, 3.0	*A* = 0.0633*C* + 0.0154	Not detectable	0.9993
Mn	0.5, 1.0, 1.5, 2.0, 2.5, 3.0	*A* = 0.0200*C* − 0.0046	0.4300	0.9988
Ni	0.25, 0.5, 1.0, 1.5, 2.0	*A* = 0.0077*C* − 0.0003	0.5584	0.9985

^∗^ *A* is absorbance, *C* is concentration (μg/L), *m* is slope, and *b* is *y*-intercept.

**Table 3:** Physicochemical qualities of the barley samples from different study areas compared to EBC standard.

Parameters	Sampling sites					EBC standard
	Beyeda	Debark	Dabat	Janamora	Wogera	
MC (%)	12.0	12.4	12.3	12.8	12.0	≤ 13.5
GE (%)	95.0	94.0	97.0	91.0	94.0	≥ 96
GC (%)	96.0	97.0	99.0	94.0	95.0	≥ 96
PC (%)	9.2	8.9	9.3	8.4	9.9	≤ 12
TKW (g)	38.1	39.5	39.1	37.2	38.7	> 30
*P* (%)	97.2	98.3	98.0	96.1	97.4	≥ 96
Sieve test (mm)						
> 2.8	43.4	68.3	45.7	29.4	45.1	MPL
> 2.5	35.2	21.6	40.2	44.6	39.1	MPL
> 2.8 + 2.5	78.6	89.9	85.9	73.9	84.2	≥ 85
> 2.2 (reject)	14.5	6.2	10.6	18.9	11.3	≤ 10

*Note: P*, purity.

Abbreviations: EBC, European Brewing Convention; GC, germination capacity; GE, germination energy; MC, moisture content; MPL, maximum permissible limit; PC, protein content; TKW, thousand kernel weight.

**Table 4:** Comparison of the physicochemical qualities of the malt sample of the present study with the European Brewing Convention (EBC) standards.

Parameters	EBC standard	This study
Sieve test		
> 2.8 + 2.5 mm	≥ 85%	93.6%
> 2.2 mm	3.0%–10%	5.1%
< 2.2 mm (reject)	Max. 1.5%	1.8%
Moisture content	3.5%–5.0%	3.7%
Extract dry matter, fine grind	77%–79%	79.1%
Extract dry matter, coarse grind	76%–77%	76.4%
Extract difference	< 2%	2.7%
Saccharification	10–15 min	10–15 min
Odor	Caramel	Caramel
Color	Not mottled	Not mottled
pH	5.6–5.8	5.7
Viscosity	1.5–1.6 cp	1.5 cp
Friability	> 80%	85%
Thousand kernel weight	> 30 g	37.6 g
Wholly unmodified grain	< 2.0%	2.4%
Partly unmodified grain	< 5.0%	2.6%

**Table 5:** Recovery test results of the analyzed metals in the barley samples (mean ± SD, *n* = 3).

Metals	Amount before spike (mg/kg)	Amount added (mg/kg)	Amount after spike (mg/kg)	Recovery (%)
Fe	139.0 ± 10.2	70.0	188.0 ± 11.2	70.0
Cu	13.0 ± 0.3	7.0	19.5 ± 0.6	92.9
Zn	20.0 ± 4.8	10.0	29.1 ± 5.2	91.0
Mn	30.0 ± 3.0	15.0	45.5 ± 5.5	103.3
Ni	11.0 ± 3.8	6.0	16.2 ± 7.2	86.7

**Table 6:** The level of the analyzed metal concentrations (mg/kg) in the barley and malt samples of the study areas.

Sample (barley)	Fe	Cu	Zn	Mn	Ni
Beyeda	133.0 ± 15.0	8.5 ± 0.6	23.0 ± 3.9	25.0 ± 1.5	15.0 ± 1.3
Janamora	95.0 ± 4.1	9.5 ± 2.31	17.0 ± 1.2	26.5 ± 0.3	9.0 ± 0.9
Debark	124.5 ± 0.6	15.0 ± 2.60	28.0 ± 2.9	24.0 ± 2.5	10.5 ± 3.8
Dabat	139.0 ± 10.2	12.5 ± 0.3	19.5 ± 4.8	30.0 ± 3.0	10.5 ± 3.8
Wogera	70.5 ± 6.8	6.5 ± 2.6	17.0 ± 2.5	27.5 ± 4.0	13.0 ± 7.5
Barley average	112.3 ± 7.3	10.4 ± 1.7	20.9 ± 3.0	26.6 ± 2.3	11.6 ± 3.4
Malt average	92.0 ± 5.9	10.7 ± 4.2	26.2 ± 5.2	26.7 ± 6.21	18.0 ± 7.5
MPL	─	3	27	2	─

Abbreviation: MPL, maximum permissible limit.

**Table 7:** Comparison of Fe, Cu, Zn, Mn, and Ni concentrations (mg/kg) in barley and malt samples of the present study with the reported literature values.

Sample	Fe	Cu	Zn	Mn	Ni	Reference
Barley	—	6.3–35.0	24.8–95.3	15.4–61.8	—	[34]
	—	1.1–11.3	20.2–23.5	4.6–16.6	—	[24]
	—	3.5–6.3	35.9–76.1	16.2–26.4	—	[25]
	—	5.2–6.9	29.4–33.3	16.7–21.4	—	[26]
	35.0–190.9	22.9–35.0	20.0–28.0	8.0–19.4	3.5–3.6	[33]
	57.5–138.5	5.5–9.9	20.0–28.0	8.0–19.4	3.4–4.2	[27]
	105.0–119.6	8.7–12.1	17.9–23.9	24.3–28.9	8.2–15.0	This study

Malt	—	3.5	10.0	24.0	—	[28]
	—	2.6	23.6	—	—	[43]
	—	5.8–6.2	—	—	—	[44]
	—	6.04	16.2	33.1	—	[36]
	77.4–82.4	0.8–0.9	27.2–32.0	27.4–35.6	1.3–1.3	[35]
	86.6–97.9	6.5–14.9	20.9–31.4	17.4–29.9	10.6–25.5	This study

## Data Availability

The data used to support the findings of this study are available from the corresponding author upon request.

## Supporting Information

Optimization conditions for sample digestion, the limit of detections, and calibration curves of the analyzed metals, and the ANOVA tables are included in the supporting information.
