# Supplementary material for: Evaluation of Physicochemical Qualities and Trace Metal Levels of Barley and Malt in North and Central Gondar Zones, Ethiopia
Source: Int J Anal Chem. 2025 Nov 6;2025:7788608. doi: 10.1155/ianc/7788608 (PMC12615037; doi:10.1155/ianc/7788608)
Supplement: Supporting Information — Additional supporting information can be found online in the Supporting Information section. [file 7788608.f1.docx]

**Evaluation of physicochemical qualities and trace metals levels of Barley and Malt in North and Central** **Gondar Zones, Ethiopia**

Mekuanint Lewoyehu^1^**^*^**, Bidir Kassaw^2^, Tadesse Bizuayehu^2^, Kenaw Gismie^3^, Ashenafei Gezahegn^4^

^1^Department of Chemistry, Bahir Dar University, Bahir Dar, P.O. Box 79, Ethiopia

^2^Department of Chemistry, Debark University, Debark, P.O. Box 90, Ethiopia

^3^Department of Chemistry, University of Gondar, Gondar, Ethiopia

^4^Department of Natural Resource Management, Debark University, Debark, Ethiopia

*Corresponding author: mekle2879@gmail.com

Supplemental Table S1. The parameters and methods/equipment used for wort analysis of the malt sample

| Parameter | Fine | coarse | Measuring method/equipment |
| --- | --- | --- | --- |
| Saccharification | 10─15 | 10─15 | Mashing bath using iodine solution |
| pH | 5.7 | 5.8 | pH meter |
| Color | 4.0 | 4.0 | Color comparator EBC method |
| Color | Not mottled | Not mottled | Naked eye |
| Odor of mash | Caramel | Caramel | Nose smell |
| Density | 1.0323 | 1.0323 | A density meter at 20 °C to determine the extract content of malt |
| Specific gravity | 1.0345 | 1.0334 |  |
| Plato (sugar) | 8.659 | 8.391 | Standard sugar table |

Supplemental Table S2. Experimental trials for determining the optimum conditions (volume ratio of the reagents, temperature, and time) for barley sample digestion to assess the level of the selected metals in the barley samples (optimum conditions are indicated in **bold**)

| Trial | Sample weight (g) | Reagent volume (mL) | | Temp  (^o^C ) | Time (hour) | Observation |
| --- | --- | --- | --- | --- | --- | --- |
|  |  | HNO_3_ | HClO_4_ |  |  |  |
| 1 | 1 | 6 | 1 | 250 | 1:45 | Clear and slightly colorless |
|  |  |  |  |  |  |  |
| 2 | 1 | 6 | 2 | 200 | 1:45 | Clear and colorless(suspension) |
| 3 | 1 | 6 | 3 | 270 | 1:45 | Clear and brownish |
| 4 | 1 | 5 | 2 | 200 | 3:15 | Clear and yellow |
| 5 | 1 | 5 | 2 | 250 | 3:30 | Clear and slightly colorless |
| 6 | 1 | 5 | 2 | 300 | 4:00 | Clear and brownish |
| **7** | **1** | **6** | **2** | **250** | **1:00** | **Clear and colorless (optimum)** |
| 8 | 1 | 6 | 2 | 250 | 1:25 | Clear and colorless |
| 9 | 1 | 6 | 2 | 250 | 2:00 | Clear and colorless |
| 10 | 1 | 4 | 1 | 270 | 3:10 | Clear and yellow |
| 11 | 1 | 5 | 1 | 270 | 3:25 | Clear and yellow |
| 12 | 1 | 6 | 1 | 270 | 4:00 | Clear and yellow |

Supplemental Table S3. Experimental trials for determining the optimum conditions (volume ratio of the reagents, temperature, and time) for malt sample digestion to assess the level of the selected metals in the malt sample (optimum conditions are indicated in bold)

| Trial | Sample weight (g) | Reagent volume  (mL) | | Temp (^o^C) | Time (hour) | Observation |
| --- | --- | --- | --- | --- | --- | --- |
|  |  | HNO_3_ | HClO_4_ |  |  |  |
| 1 | 1 | 4 | 1 | 200 | 3:30 | Clear and yellow |
| 2 | 1 | 4 | 2 | 250 | 3:30 | Clear and yellow |
| 3 | 1 | 4 | 3 | 300 | 3:30 | Clear and yellow |
| 4 | 1 | 5 | 1 | 250 | 2:30 | Clear and slightly colorless |
| 5 | 1 | 5 | 1 | 250 | 3:00 | Clear and slightly colorless |
| 6 | 1 | 5 | 1 | 250 | 3:25 | Clear and brownish |
| 7 | 1 | 6 | 2 | 200 | 2;25 | Clear and colorless |
| 8 | 1 | 6 | 2 | 200 | 2:30 | Clear and colorless |
| **9** | **1** | **6** | **2** | **200** | **2:15** | **Clear and colorless (optimum)** |
| 10 | 1 | 4 | 1 | 300 | 1:30 | Clear and yellow |
| 11 | 1 | 3 | 1 | 270 | 3:25 | Clear and yellow |
| 12 | 1 | 2 | 1 | 270 | 4:00 | Clear and yellow |

Supplemental Table S4. The Limit of Detection (LOD) and Limit of Quantification (LOQ) of the Flame Atomic Absorption Spectrophotometer (FAAS) for each metal determined in the barley and malt samples

| Metals | Fe | Cu | Zn | Mn | Ni |
| --- | --- | --- | --- | --- | --- |
| LOD (µg/L) | 0.236 | 3.180 | 2.040 | 0.531 | 0.042 |
| LOQ (µg/L) | 0.741 | 8.520 | 5.970 | 1.430 | 0.121 |

Supplemental Table S5. Barley sample weights before and after drying for moisture content determination at the five sampling sites*—*Beyeda (Bey), Janamora (Jan), Debark (Deb), Dabat (Dab), and Wogera (Wog)

| Districts | Plate No. | Plate  Weight (g) | Sample  Weight (g) | TWBD  (g) | TWAD  (g) | MC (%) |
| --- | --- | --- | --- | --- | --- | --- |
| Bey | 1 | 5.412 | 4.5 | 9.912 | 9.380 | 11.8 |
| Jan | 2 | 5.416 | 4.5 | 9.916 | 9.348 | 12.6 |
| Deb | 3 | 5.439 | 4.5 | 9.939 | 9.382 | 12.4 |
| Dab | 4 | 5.444 | 4.5 | 9.944 | 9.401 | 12.1 |
| Wog | 5 | 5.420 | 4.5 | 9.920 | 9.386 | 11.9 |
| TWBD: total weight before drying; TWAD: total weight after drying; MC: Moisture content | | | | | | |

**Summary of ANOVA results for the analyzed metals in the barley samples**

Supplemental Table S6. Summary of ANOVA results for the content of iron (Fe) in barley samples across the five sampling sites—Beyeda (Bey), Janamora (Jan), Debark (Deb), Dabat (Dab), and Wogera (Wog)

| Groups | Count | Sum | Average | Variance |  |  |
| --- | --- | --- | --- | --- | --- | --- |
| Fe - Bey | 3 | 7.97 | 2.656667 | 0.093633333 |  |  |
| Fe - Jan | 3 | 5.71 | 1.903333 | 0.006533333 |  |  |
| Fe- Deb | 3 | 7.46 | 2.486667 | 0.000133333 |  |  |
| Fe- Dab | 3 | 8.37 | 2.79 | 0.0412 |  |  |
| Fe- Wog | 3 | 4.24 | 1.413333 | 0.018233333 |  |  |
| Source of variation | SS | df | MS | F | P-value | F crit |
| Between groups | 3.999533 | 4 | 0.999883 | 31.29851836 | 1.25E-05 | 3.47805 |
| Within groups | 0.319467 | 10 | 0.031947 |  |  |  |
| Total | 4.319 | 14 |  |  |  |  |

Supplemental Table S7. Summary of ANOVA results for the content of copper (Cu) in barley samples across the five sampling sites —Beyeda (Bey), Janamora (Jan), Debark (Deb), Dabat (Dab), and Wogera (Wog)

|  | Groups | Count | Sum | Average | Variance |  |  |
| --- | --- | --- | --- | --- | --- | --- | --- |
|  | Cu -Bey | 3 | 0.5 | 0.166667 | 0.000133 |  |  |
|  | Cu -Jan | 3 | 0.56 | 0.186667 | 0.002133 |  |  |
|  | Cu-Deb | 3 | 0.9 | 0.3 | 0.0027 |  |  |
|  | Cu-Dab | 3 | 0.74 | 0.246667 | 3.33E-05 |  |  |
|  | Cu-Wog | 3 | 0.39 | 0.13 | 0.0027 |  |  |
|  | Source of Variation | SS | df | MS | F | P-value | F crit |
|  | Between Groups | 0.05456 | 4 | 0.01364 | 8.857143 | 0.002532 | 3.47805 |
|  | Within Groups | 0.0154 | 10 | 0.00154 |  |  |  |
|  | Total | 0.06996 | 14 |  |  |  |  |

Supplemental Table S8. Summary of ANOVA results for the content of zinc (Zn) in barley samples across the five sampling sites*—*Beyeda (Bey), Janamora (Jan), Debark (Deb), Dabat (Dab), and Wogera (Wog)

| Groups | Count | Sum | | Average | Variance |  |  |
| --- | --- | --- | --- | --- | --- | --- | --- |
| Zn-Bey | 3 | 1.39 | 0.463333 | | 0.006033 |  |  |
| Zn -Jan | 3 | 1.03 | 0.343333 | | 0.000533 |  |  |
| Zn-Deb | 3 | 1.69 | 0.563333 | | 0.003233 |  |  |
| Zn-Dab | 3 | 1.17 | 0.39 | | 0.0091 |  |  |
| Zn-Wog | 3 | 1.02 | 0.34 | | 0.0025 |  |  |
| Source of Variation | SS | df | MS | | F | P-value | F crit |
| Between groups | 0.1068 | 4 | 0.0267 | | 6.238318 | 0.008758532 | 3.47805 |
| Within groups | 0.0428 | 10 | 0.00428 | |  |  |  |
| Total | 0.1496 | 14 |  | |  |  |  |

Supplemental Table S9. Summary of ANOVA results for the content of manganese (Mn) in barley samples across the five sampling sites *—*Beyeda (Bey), Janamora (Jan), Debark (Deb), Dabat (Dab), and Wogera (Wog)

| Groups | Count | Sum | Average | Variance |  |  |
| --- | --- | --- | --- | --- | --- | --- |
| Mn-Bey | 3 | 1.49 | 0.496667 | 0.000833 |  |  |
| Mn -Jan | 3 | 1.6 | 0.533333 | 3.33E-05 |  |  |
| Mn-Deb | 3 | 1.44 | 0.48 | 0.0025 |  |  |
| Mn-Dab | 3 | 1.79 | 0.596667 | 0.003333 |  |  |
| Mn-Wog | 3 | 1.64 | 0.546667 | 0.005833 |  |  |
| Source of Variation | SS | df | MS | F | P-value | F crit |
| Between Groups | 0.025027 | 4 | 0.006257 | 2.496011 | 0.10975 | 3.47805 |
| Within Groups | 0.025067 | 10 | 0.002507 |  |  |  |
| Total | 0.050093 | 14 |  |  |  |  |

Supplemental Table S10. Summary of ANOVA results for the content of nickel (Ni) in barley samples across the five sampling sites*—*Beyeda (Bey), Janamora (Jan), Debark (Deb), Dabat (Dab), and Wogera (Wog)

| Groups | Count | Sum | Average | Variance |  |  |
| --- | --- | --- | --- | --- | --- | --- |
| Ni-Bey | 3 | 0.9 | 0.3 | 0.0507 |  |  |
| Ni -Jan | 3 | 0.54 | 0.18 | 0.0003 |  |  |
| Ni-Deb | 3 | 0.64 | 0.213333 | 0.005633 |  |  |
| Ni-Dab | 3 | 0.64 | 0.213333 | 0.005633 |  |  |
| Ni-Wog | 3 | 0.77 | 0.256667 | 0.022533 |  |  |
| Source of Variation | SS | df | MS | F | P-value | F crit |
| Between Groups | 0.025893 | 4 | 0.006473 | 0.381682 | 0.816897252 | 3.47805 |
| Within Groups | 0.1696 | 10 | 0.01696 |  |  |  |
| Total | 0.195493 | 14 |  |  |  |  |

Supplemental Figure S1. Instrumental (FAAS) calibration curves obtained from absorbance readings of five series of standard solutions of the analyzed metals: A. Iron (Fe), B. Copper (Cu), C. Zinc (Zn), D. Manganese (Mn), E. Nickel (Ni)

A
